# Overexpression of *Scg5 *increases enzymatic activity of PCSK2 and is inversely correlated with body weight in congenic mice

**DOI:** 10.1186/1471-2156-9-34

**Published:** 2008-04-25

**Authors:** Charles R Farber, James Chitwood, Sang-Nam Lee, Ricardo A Verdugo, Alma Islas-Trejo, Gonzalo Rincon, Iris Lindberg, Juan F Medrano

**Affiliations:** 1 Department of Animal Science, University of California, Davis, One Shields Ave., Davis, CA 95616-8521, USA; 2 Department of Biochemistry and Molecular Biology, Louisiana State University Health, Sciences Center, and Children's Hospital Research Institute, New Orleans, LA 70112-2223, USA; 3 Department of Medicine, Division of Cardiology, University of, California, Los Angeles, 695 Charles E. Young Drive South, Los Angeles, CA 90095-1679, USA; 4 Research Center for Human Natural Defense System, Yonsei, University College of Medicine, Seoul, 120-752, Korea; 5 Department of Anatomy and Neurobiology, University of, Maryland Medical School, 20 Penn St, Baltimore, MS 21201, USA

## Abstract

**Background:**

The identification of novel genes is critical to understanding the molecular basis of body weight. Towards this goal, we have identified secretogranin V (*Scg5*; also referred to as *Sgne1*), as a candidate gene for growth traits.

**Results:**

Through a combination of DNA microarray analysis and quantitative PCR we identified a strong expression quantitative trait locus (eQTL) regulating *Scg5 *expression in two mouse chromosome 2 congenic strains and three additional F2 intercrosses. More importantly, the eQTL was coincident with a body weight QTL in congenic mice and *Scg5 *expression was negatively correlated with body weight in two of the F2 intercrosses. Analysis of haplotype blocks and genomic sequencing of *Scg5 *in high (C3H/HeJ, DBA/2J, BALB/cByJ, CAST/EiJ) and low (C57BL/6J) expressing strains revealed mutations unique to C57BL/6J and possibly responsible for the difference in mRNA abundance. To evaluate the functional consequence of *Scg5 *overexpression we measured the pituitary levels of 7B2 protein and PCSK2 activity and found both to be increased. In spite of this increase, the level of pituitary α-MSH, a PCSK2 processing product, was unaltered.

**Conclusion:**

Together, these data support a role for *Scg5 *in the modulation of body weight.

## Background

Body weight, as with all complex traits, is partially regulated by the coordinate action of individual genes. One common approach for dissecting the genetics of growth is the mapping of quantitative trait loci (QTL). In the last decade numerous human and mouse growth QTL have been identified [1]; however, while this progress is important, few if any of the loci have been unequivocally resolved to the effects of a single quantitative trait gene (QTG).

Several studies involving many different mouse inbred strains have demonstrated an enrichment of growth and obesity QTL on chromosome 2 [2,3]. In our laboratory, we have developed a number of genomic resources with the goal of discerning the molecular basis of chromosome 2 QTL segregating between the C57BL/6J (B6), C57BL/6J-*hg/hg *(HG) and CAST/EiJ (CAST) strains [4,5]. These include two congenic strains, B6.CAST-(*D2Mit329-D2Mit457*)N(6) (B62D) and HG.CAST-(*D2Mit329-D2Mit457*)N(6) (HG2D), constructed by introgressing an identical congenic donor region, extending from approximately 75 to 180 Mbp, from the CAST strain onto both B6 and HG backgrounds [4]. HG mice exhibit extreme body weights without becoming obese due to the *high growth *(*hg*) deletion located on chromosome 10, which eliminates the expression of the suppressor of cytokine signaling 2 (*Socs2*) gene, a negative regulator of growth hormone signaling [6]. The purpose of generating the HG2D and B62D strains was to capture the weight gain 2 (*Wg2*) QTL [7] and evaluate hypothesized interactions between *Wg2 *and *hg*. After construction, HG2D mice were not characterized due to reproductive problems, however, B62D mice displayed significant decreases in growth and obesity traits [4].

In two follow-up studies it was determined that *Wg2 *was actually the result of multiple genes. In the first study, an F2 cross derived from the HG2D congenic and HG progenitor strain (referred to as the HG2DF2 cross) was used to identify a strong QTL located at 112 Mbp that decreased body weight at 6 and 9 weeks of age, but did not affect fat mass. This QTL partially explained the effects of *Wg2 *and was referred to as weight gain QTL 5 (*Wg5*) [5]. In the second study, subcongenic mice derived from the B62D congenic (referred to as B62D-3), with a CAST donor region from 102–115 Mbp, were lighter and leaner due to the effects of a locus we named *Wg2b *[8]. Based on their coincident genomic location, *Wg5 *and *Wg2b *are likely the same QTL, however, their differing effects on adiposity suggest that an interaction with *hg *may eliminate or decrease the fat reducing effects of *Wg5*.

In the current study we have used DNA microarrays and public genomic databases to investigate the genetics of expression for genes located within the two congenic models, HG2D and B62D, to identify candidates for the *Wg5 *and *Wg2b *QTL. Based on its genomic location, differential expression and biological function, the most promising candidate is secretogranin V (*Scg5*; also referred to as *Sgne1*). The protein product of *Scg5*, 7B2, is a molecular chaperone for prohormone convertase 2 (PCSK2). PCSK2 is a member of the subtilisin-like proprotein convertase family and many PCSK2-generated peptides are either directly involved in glucose homeostasis (glucagon) or indirectly contribute to body weight regulation (α-MSH, CART, and nesfatin-1, to name a few examples). Knockout of peptide biosynthetic enzymes often result in mice with body weight phenotypes, most notably the *fat/fat *mouse with its inactivating mutation in *carboxypeptidase E *[9]. While the PCSK2 knockout mouse is slightly runted but otherwise of normal weight [10], an obese PCSK1 mutant mouse has recently been described which has lowered levels of the PCSK2 product α-MSH [11], indicating possible interplay/feedback between the peptide synthesizing enzymes (also suggested by [12]). *Scg5 *has also been implicated in body weight homeostasis. *Scg5 *knockout mice (on a 129/SvEv background) exhibit a postnatal lethal phenotype in which all mice die by 5 weeks of age (due to intermediate lobe ACTH hypersecretion and rampant corticosteronemia); however, adrenalectomy rescues these animals and reveals a late-onset obesity phenotype [13]. Collectively, these results point to potential roles for peptide biosynthetic enzymes, and specifically for *Scg5*, in body weight regulation.

## Results

### Identification of differentially expressed genes in HG2DF2 mice

To identify differentially expressed genes within the HG2D congenic donor region that potentially underlie body weight and obesity QTL, we hybridized whole brain RNA from non-recombinant HG2DF2 mice (mice inheriting intact *cast *or *b6 *congenic haplotypes) from each of the three F2 genotypes (*b6/b6*, *b6*/*cast *and *cast/cast*) [5] to Agilent whole genome mouse microarrays. Comparison of normalized expression values identified 62 genes as differentially expressed at a false discovery rate (FDR) of <0.30 using an additive effects model (Additional File 1). Fifty-five of the genes (88.7%) were located within the HG2D congenic region (from approximately 75 to 180 Mbp).

### Identification of expression QTL (eQTL) overlapping growth and obesity *QTL*

As confirmation of the microarray results, we next examined the genetic basis of expression for eleven genes (*Cd44*, *AI451465*, *Fmn*, *Scg5*, *2310032D16Rik*, *3300001M20Rik*, *Pak7*, *Actr5*, *D930001I22Rik*, *Sdccag33l *and *Rab22a*) identified as differentially expressed from the microarray study (Additional File 1). These genes were selected for confirmation because they were located near the peaks of previously identified HG2DF2 body weight and obesity QTL (Table 1) [5]. Gene expression was measured in whole brain RNA from 45 recombinant HG2DF2 mice using quantitative PCR. Of the eleven genes, significant eQTL were identified for five (*AI451465*, *Scg5*, *2310032D16Rik*, *3300001M20Rik *and *Actr5*) (Table 1).

**Table 1:** Expression QTL for genes coincident with growth or obesity QTL and differentially expressed in HG2D mice

Gene symbol	Mbp	eQTL LOD	cM	Mbp	Coincident QTL
*AI451465*	112.272	9.8	64.0	134	*Wg5*
*Scg5*	113.578	16.4	55.5	115	*Wg5*
*2310032D16Rik*	132.221	5.5	55.5	115	*Fatq1*
*3300001M20Rik*	132.496	3.0	66.5	138	*Fatq1*
*Actr5*	158.316	8.6	75.0	155	*Fatq1, Fatq2*

All eQTL are significant at P < 0.05

### Expression of Scg5 is genetically regulated by a single local acting QTL in multiple mouse crosses

**Figure 1:**
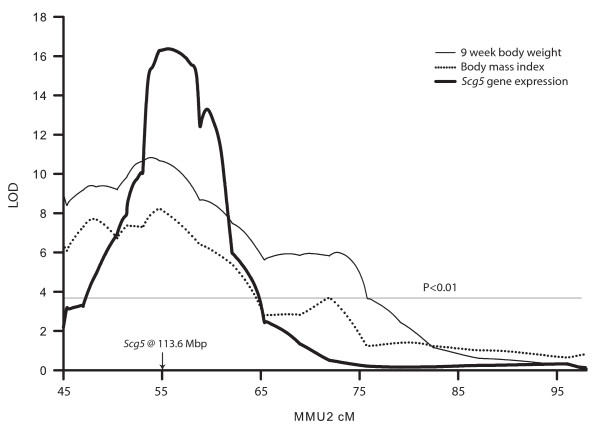
***Scg5 *****expression is regulated by a strong local eQTL on chromosome 2 and is coincident with QTL for body weight and body mass index in HG2DF2 mice.** The plot presents the LOD score profile across the HG2D donor region for *Scg5 *expression, body weight at 9 weeks and body mass index. Genetic positions in centimorgan (cM) are plotted on the x-axis. The location of *Scg5 *(113.6 Mbp) is indicated with an arrow.

Based on its close proximity to *Wg5 *[5], biological function and magnitude of differential expression, *Scg5 *was selected as the highest priority candidate gene. From the microarray analysis, *Scg5 *was the eighth most significantly differentially expressed gene, with an FDR = 0.10, and its expression was up-regulated by approximately 3.0 fold in non-recombinant *cast/cast *HG2DF2 mice (Additional File 1). qPCR analysis in HG2DF2 mice indicated the expression difference was due to a *Scg5 *eQTL located at 55.5 cM (approximately 115 Mbp) mapping with a maximum LOD score of 16.4 (Figure 1). The eQTL was primarily additive (a = -0.68 and d = -0.14), with *cast *alleles increasing expression. The eQTL explained 74.1% of the variation in *Scg5 *expression. The nearest marker, *D2Mit207 *(located at 9.7 cM or 111.8 Mbp), was less than 2 Mbp from the physical location of *Scg5 *(113.6 Mbp), strongly suggesting the variation controlling the difference in expression was local in nature (eQTL have traditionally been referred to as *cis *if the variation affecting expression lies in the differentially expressed gene as is the case for *Scg5*, however, true *trans *eQTL may also show a similar behavior. Therefore, we have adopted the use of the more general local and distant nomenclature recently proposed by [14]). Importantly, previously identified QTL for body weight at 9 weeks (*Wg5*) [5] and body mass index in HG2DF2 mice (located at 54 cM and 54.7 cM, respectively) were coincident with the *Scg5 *eQTL (Figure 1).

**Figure 2:**
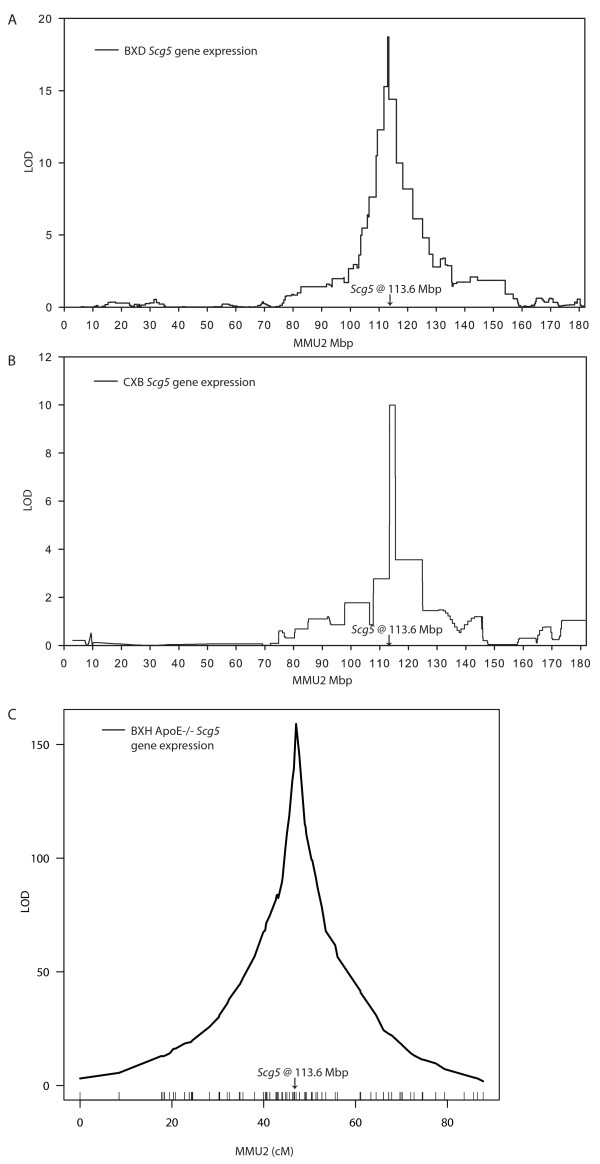
**A strong local eQTL on chromosome 2 regulates the expression of *Scg5 *in BXD, CXB and BXH mouse crosses.** Mapping of *Scg5 *expression in A) whole brain from B6 × DBA (BXD) recombinant inbred (RI) mice, B) hippocampus from BALB × B6 (CXB) RI mice, and C) whole brain from BXH-*ApoE*^-/- ^F2 mice. eQTL were identified using interval mapping tools available at [15, 16] for BXD and CXB crosses and R/qtl [38] software for BXH-*ApoE*^-/- ^F2 mice.

To determine the effect of genetic background on transcriptional regulation of *Scg5*, we performed QTL analysis with expression data from three different crosses. Two were analyzed using the GeneNetwork resource for system genetics [15,16]. The GeneNetwork contains microarray data on whole brain gene expression in B6 × DBA (BXD) recombinant inbred (RI) strains [17] and hippocampus gene expression in B6 × BALB (CXB) RI strains. QTL analysis for the levels of *Scg5 *expression revealed strong local eQTL in both crosses (Figure 2). *Scg5 *expression was also found to be under the control of a strong local eQTL using whole brain expression data from a recently described B6-*ApoE*^-/- ^× C3H-*ApoE*^-/- ^F2 intercross (BXH-*ApoE*^-/- ^F2) (Figure 2) [18]. In all three crosses B6 alleles decreased expression. Additionally, the expression of *Scg5 *was correlated with body weight in BXD and CXB mice, but not in the BXH-*ApoE*^-/- ^F2 cross (Table 2). Thus, genetic variation near the *Scg5 *locus accounts for the majority of variation in mRNA levels in multiple genetic backgrounds, and importantly the low-expressing allele is unique to B6.

**Table 2:** Correlation between *Scg5 *expression and body weight in five mouse crosses

Cross	Trait	Correlation	p-value	Details
HG2DF2	9 week weight	-0.34	0.02	calculated in 45 HG2DF2 mice
B62D-3	6 week weight	-0.45	0.01	calculated in 15 non-recombinant B62D-3 mice, 5 of each genotype
B62D-3	9 week weight	-0.62	<0.01	calculated in 15 non-recombinant B62D-3 mice, 5 of each genotype
BXD RI	Adult body weight	-0.53	0.02	calculated in 16 BXD RI and 2 parental strains, phenotype = BXHPublish:10031 [15, 16]
CXB RI	90 day body weight	-0.82	<0.01	calculated in 7 BXD RI and 2 parental strains, phenotype = BXHPublish:10281 [15, 16]
BXH-*ApoE*^-/- ^F2	Adult body weight	-0.01	0.81	calculated in 255 BXH-*ApoE*^-/- ^F2 mice

### Transcriptional regulation of Scg5 in B62D-3 F2 mice

**Figure 3:**
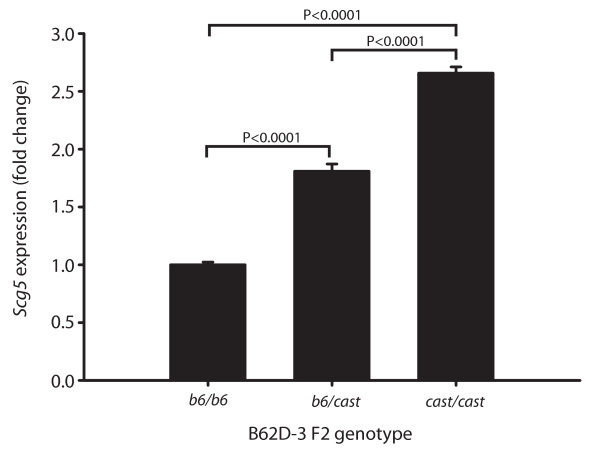
***Scg5*****expression is up-regulated in B62D-3 F2 mice.** The relative expression of *Scg5 *is presented for the three B62D-3 F2 genotypes (*b6*/*b6*, *b6*/*cast *and *cast/cast*).

Since *Scg5 *is regulated by a strong local eQTL in HG2DF2 mice (Figure 1) we also expected it to be differentially expressed in wild type B62D-3 F2 mice. B62D-3 mice are wild-type with respect to the *hg *deletion and are congenic for *cast *alleles from 102–115 on chromosome 2 in the region of *Scg5 *[8]. To confirm differential expression we measured mRNA levels in mice of all three B62D-3 F2 genotypes (*b6/b6*, *b6/cast *and *cast/cast*). As expected, *Scg5 *was up-regulated in *cast*/*cast *mice by approximately 2.5 fold (Figure 3). Additionally, if differential expression of *Scg5 *is causal for *Wg5 *(in HG2DF2 mice) and *Wg2b *(in B62D-3 mice), then its expression must correlate with growth traits and indeed its expression was significantly negatively correlated (P < 0.01) with body weight and obesity in both crosses (Table 2).

### Scg5 expression is up-regulated in high growth (HG) mice

If an interaction exists between *Wg5 *and *hg *(as has been hypothesized in [4,7]), it may be mediated via alterations in *Scg5 *expression. To determine if expression is altered by *hg*, *Scg5 *was quantified in HG and B6 mice at 3, 4.5 and 9 weeks of age. At all three time points the relative expression of *Scg5 *in whole brain was increased in HG mice (Additional File 2). A suggestive (P < 0.10) 25% increase at 3 weeks was followed by approximately 40% increases at 4.5 (P < 0.05) and 9 weeks (P = 0.06).

### Identification of Stat5b binding sites in the Scg5 promoter

The *hg *deletion encompasses three genes; *Socs2*, *Raidd *and *Plexin C1 *[19]. The extreme growth rate and mature body size of HG mice (homozygous for the *hg *deletion) is primarily (if not entirely) due to the absence of *Socs2 *[6]. In *Socs2*^-/- ^mice increased growth is dependent on *Stat5b *[20]. The DNA sequence required for *Stat5b *binding has been well characterized [21].*Stat5b *is the primary transcription factor responsible for mediating *Gh *induced gene expression changes [22] and a modest increase in its activity has been identified in *Socs2*^-/- ^mice [20]. To identify putative *Stat5b *binding sites which may be used by *Gh *(via *Stat5b*) to regulate the increase of *Scg5 *expression in HG mice, we screened 72.683 Kbp of *Scg5 *genomic sequence for the presence of the *Stat5b *consensus transcription factor binding site TTCYNRGAA (Y = T or C and R = A or G) [21]. The sequence included all introns and exons and 10 kbp of 5' and 3' genomic sequence. In total six consensus sites were identified (Additional File 2). Interestingly, two tandem sites in the *Scg5 *promoter were located between -56 and -39 (relative to the transcription start site; Additional File 2). The probability that these sites occur by chance is 4^18^, suggesting they are functional and may mediate the increased expression of *Scg5 *in HG mice.

### Genomic analysis of the Scg5

It is likely that most polymorphisms giving rise to local (truly *cis*) eQTL will reside within promoter or intronic based regulatory elements [23]. With this in mind, we analyzed the genomic sequence of *Scg5 *to identify putative polymorphisms that may be responsible for the strong local eQTL. This is important since it could lead to the discovery of novel modes of *Scg5 *transcriptional regulation. Based on the eQTL results from multiple crosses, we hypothesized that two haplotypes should exist in the genomic region containing the eQTL, one unique to B6 (low expressers) the other shared between CAST, BALB, DBA and C3H (high expressers). To identify potential regions, haplotype blocks were identified by downloading all SNPs within *Scg5 *from the Mouse Phenome Database [24,25]. A total of 191 SNPs were identified between 113.571079 and 113.633403 Mbp on chromosome 2 (Additional File 3). This 62.324 Kbp region encompassed the entire *Scg5 *coding sequence, promoter, over 5 Kbp downstream and the 3' end of *Arhgap11a*, which is located 2.4 kbp upstream of the first *Scg5 *exon. As predicted, two haplotype blocks were identified using the Haploview software package [26]. Block 1 extended from the beginning of the queried region at 113.571079 Mbp to 113.581978 Mbp (10.899 Kbp) and block 2 spanned from 113.582743 Mbp to the end of the interval at 113.633403 Mbp (50.660 Kbp) (Additional File 3). Block 1 did not fit the necessary criteria of being unique to B6 (B6 and CAST were very similar across this block), however, block 2 consisted of three predominant haplotypes, one unique to B6, one unique to CAST and the other was highly similar among C3H, DBA and BALB. Although CAST differed from all other strains across the whole block, it was much more similar to C3H, DBA and BALB in the region corresponding to exon 2 and the first two introns. The haplotype similarities and differences across both blocks are shown in cladograms in Additional File 4. Therefore, since the region of haplotype block 2 contains a haplotype unique to B6 and similar among the other strains it is likely the genetic variant underlying the eQTL is located in this region.

None of the SNPs included in the above analysis were located within the proximal promoter. To comprehensively screen this region, we sequenced 2500 bps upstream of the transcription start site (as defined by RefSeq NM_009162) in B6, HG, CAST, BALB, DBA and C3H mice. Ten SNPs were identified across all six strains (Table 3). In addition, an 11 bp deletion between -432 and -422 (del-432) was identified in DBA, C3H and BALB. This deletion was previously identified in C3H mice by Schmidt *et al*. [27]. Consistent with the above haplotype analysis, BALB, DBA and C3H shared an identical haplotype across the region. CAST was polymorphic at all 10 of the SNPs (but not del-432) relative to B6 and the other strains were polymorphic at SNPs -1373, -794 and -659. Interestingly, CAST mice did not possess del-432, however, position -432 was polymorphic between B6 and CAST mice. Therefore, since these four SNPs are unique to B6, the low *Scg5 *expressing strain, it is probable that one (or more) may give rise to the *Scg5 *eQTL

**Table 3:** *Scg5 *promoter polymorphisms in C57BL/6J, C57BL/6J-*hg/hg*, BALB/cByJ, C3H/HeJ, DBA/2J and CAST/EiJ strains

Strain	*Scg5 *exp.	-2475 (A/G)	-2445 (A/C)	-1540 (A/G)	-1373 (A/G)	-1095 (G/C)	-904 (G/T)	-794 (G/A)	-659 (A/G)	-432 (C/T)	del-432	-306 (A/C)
C57BL/6J	LOW	AA	AA	AA	GG	GG	GG	GG	AA	CC	+	AA
C57BL/6J-*hg/hg*	LOW	AA	AA	AA	GG	GG	GG	GG	AA	CC	+	AA
BALB/cByJ	HIGH	AA	AA	AA	***AA***	GG	GG	***AA***	***GG***	***del***	del	N/A
C3H/HeJ	HIGH	AA	AA	AA	***AA***	GG	GG	***AA***	***GG***	***del***	del	N/A
DBA/2J	HIGH	AA	AA	AA	***AA***	GG	GG	***AA***	***GG***	***del***	del	N/A
CAST/EiJ	HIGH	GG	CC	GG	***AA***	CC	TT	***AA***	***GG***	***TT***	+	CC

SNPs are numbered based on position upstream from transcription start site as defined by RefSeq NM_009162,. SNPs with genotypes in bold italics segregate low expressers and high expressers. +, deletion not present in these strains; del, deletion is present in these strains; N/A, genotypes for -306 were not determined for strains with deletion due to the forward primer sequence overlapping the deletion.

### Up-regulation of Scg5 increases levels of 7B2 protein and PCSK2 enzyme activity

**Figure 4:**
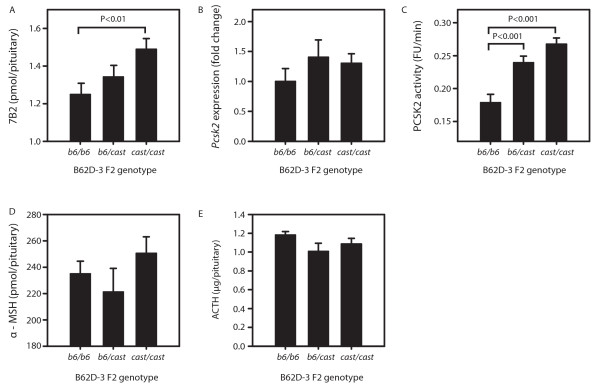
**7B2 levels and PCSK2 activity are increased in B62D-3 F2 mice overexpressing *Scg5*.** A) 7B2 pituitary levels, B) *Pcsk2 *mRNA expression, C) PCSK2 activity, D) pituitary α-MSH and E) pituitary ACTH levels in the three B62D-3 F2 genotypes (*b6*/*b6*, *b6*/*cast *and *cast/cast*).

To characterize the molecular consequences of *Scg5 *up-regulation, the levels of 7B2 protein, *Pcsk2 *gene expression, PCSK2 activity, α-MSH and ACTH levels were quantitated in B62D-3 F2 mice. The levels of pituitary (pit) 7B2 mirrored the differences in whole brain *Scg5 *expression in mice of differing B62D-3 genotype. *b6/b6 *mice had the lowest levels with 1.25 ± 0.04 pmol/pit, *b6*/*cast *mice followed with 1.34 ± 0.04 pmol/pit while *cast*/*cast *mice had the highest levels of 1.55 ± 0.04 pmol/pit (Figure 4). The difference between *b6/b6 *and *cast*/*cast *mice was highly significant (P < 0.01). In addition, the activity of PCSK2 also increased coincident with *Scg5 *mRNA abundance and 7B2 protein levels (Figure 4). The difference in activity between *b6/b6 *and *cast*/*cast *genotypes was 27%. All pairwise genotype comparisons were highly significant (P < 0.0001). The distal border of B62D-3 ends at 115 Mbp and *Pcsk2 *is located on chromosome 2 at 143 Mbp. Therefore, the difference in PCSK2 activity could not result from CAST and B6 allelic differences at the *Pcsk2 *locus. To rule out distant-regulation of *Pcsk2 *in B62D-3 mice we measured *Pcsk2 *mRNA levels. No difference in expression was observed due to B62D-3 genotype (Figure 4). α-MSH and ACTH are secreted peptides, both products of convertase-mediated proopiomelanocortin processing in the pituitary. Although PCSK2 activity was increased, no differences were seen in the tissue levels of either peptide (Figure 4).

## Discussion

In the current study we have demonstrated that transcriptional variation in *Scg5 *across several inbred strains is mediated almost entirely by local genetic variation near the *Scg5 *locus. More importantly, we have demonstrated that an increase in *Scg5 *expression is correlated with decreases in body weight and obesity in two congenic mouse models and overexpression results in an *in vivo *increase in 7B2 protein levels and PCSK2 enzymatic activity.

By analyzing the expression of *Scg5 *in multiple segregating populations we have conclusively shown that nearly all of the variation in *Scg5 *expression is due to a strong local-acting variant. In all four crosses surveyed, the eQTL accounted for over 75% of the variation in *Scg5 *expression. Haplotype analysis using a recently generated high-density SNP collection indicated that the most probable location for the *Scg5 *local eQTL quantitative trait nucleotide was the promoter through the first four exons/introns. In the *Scg5 *promoter, four polymorphisms unique to the high-expressing strains were discovered. Of particular interest is del-432, an 11-bp deletion present in all strains except B6 and CAST. Although the deletion is absent in CAST, these mice are polymorphic at position -432 (possessing a T allele instead of the C allele in B6). It is tempting to speculate that this region is important for binding of a transcriptional repressor and that -432 is a key nucleotide.

The action of *Scg5 *overexpression on body weight may represent either a direct effect of 7B2 or an indirect effect mediated by PCSK2-generated peptides. In this regard, it should be pointed out that it is not yet clear that 7B2 is always regulatory for PCSK2-mediated peptide production. A recent paper showing that the varying 7B2 levels synthesized by two different mouse strains are associated with differential production of glucagon [27] supports the notion that 7B2 levels do modulate *in vivo *peptide production, possibly by regulation of PCSK2 activity. However, our present results do not support an obligatory association of increased peptide production by increased PCSK2 activity, since we observed no changes in pituitary α-MSH, a PCSK2 product, even though tissue PCSK2 activity increased. While two studies have shown that introduction of 7B2 into cells which lacks all 7B2 production facilitates the generation of PCSK2-mediated peptides [28,29], when cells already express some *Scg5*, additional 7B2 introduction does not always increase peptide production. An example is the transfection of *Scg5 *into AtT-20-PC2 cells, a neuroendocrine cell line which endogenously produces 7B2. The additional increase of 7B2 neither increases α-MSH production nor facilitates any other PCSK2-mediated peptide cleavage examined to date [30] and SNL and IL, unpublished results). Presumably other factors, possibly cell- or tissue-specific, can override any effects of additional *Scg5 *expression to limit the total amount of peptide made. In this regard it is important to note that we measured PCSK2-mediated peptide production only in pituitary; PCSK2/7B2 effects may also occur in other tissues more intimately involved in energy homeostasis, such as the hypothalamus.

If the action of 7B2 on body weight homeostasis is not mediated via generation of PCSK2-mediated peptides, then it may result from a direct effect of 7B2 itself. It is currently impossible to speculate on the mechanism for this effect; however, this interesting pan-neuronal protein may have many unexplored roles. A recent paper describes the association of human neuroblastomas with the epigenetic down-regulation of 7B2 [31]. In this study, loss of 7B2 expression was associated with increased tumor formation, suggesting that 7B2 expression may contribute to the maintenance of neuronal differentiation.

Although we have provided several lines of evidence supporting the role of *Scg5 *in body weight regulation, that is in agreement with a prior knockout study demonstrating an increase in body weight in the absence of *Scg5 *[13], we have not demonstrated a direct causal link between *Scg5 *overexpression and reductions in body weight. It is possible that the local eQTL, regulating *Scg5 *expression, is in tight linkage with the actual causal variant(s) responsible for *Wg5*. It will take additional studies, such as generating a *Scg5 *transgenic mouse to confirm its involvement in body weight regulation.

In the current study we have shown that in addition to the effects of the strong eQTL, *Scg5 *is up-regulated in HG mice, suggesting that this gene is responsive to growth hormone signaling. As discussed above, HG mice lack expression of *Socs2*. The absence of *Socs2 *leads an increase in the activity of *Stat5b *in *Socs2*^-/- ^mice a factor which mediates the transcriptional effects of growth hormone [20]. The molecular and physiological consequences of this increase are unclear; however, it should be noted two previous studies have provided data supporting an interaction between *hg *and chromosome 2 QTL [4,7]. It is possible that the increase in *Scg5 *expression underlies this interaction.

## Conclusion

We have provided data to support *Scg5 *as a candidate gene for body weight homeostasis. Further, our results show that expression of the *Scg5 *gene product, 7B2, is likely to be controlled by strain-dependent promoter polymorphisms and that this difference in expression leads to an increase in the enzymatic activity of PCSK2. The mechanism by which increased 7B2 levels contribute to body weight is unclear as yet, but may represent a PC2-independent effect of this widely-expressed neuroendocrine gene.

## Methods

### Mice

Phenotypic and gene expression data were generated from two inbred strains (B6 and HG [32]), a subcongenic strain (B62D-3 [8]) and two congenic-derived intercrosses (HG2DF2 [5] and B62D-3 F2 [8]). In addition, *Scg5 *sequence data were collected from four inbred strains C3H/HeJ (C3H), DBA/2J (DBA), BALB/cByJ (BALB) and CAST and *Scg5 *expression data were obtained from three additional crosses not produced in our laboratory, the BXD RI, CXB RI and BXH-*ApoE-/- *F2 [16,18]. All animal protocols were managed according to the guidelines of the American Association for Accreditation of Laboratory Animal Care (AAALAC).

### Microarray gene expression analysis in whole brain RNA from non-recombinant HG2DF2 mice

Total RNA was isolated using TRIzol reagent (Invitrogen) from homogenized whole brain samples. Whole brain total RNA from four male non-recombinant (*hg*/*hg*, *hg*/*cast *or *cast*/*cast *across the entire congenic interval) mice was used for microarray analysis. Two samples from each genotype were labeled with each dye (Cy3 or Cy5) using the Agilent Low RNA Input Fluorescent Linear Amplification Kit (Agilent Technologies). Samples were hybridized on the Mouse Whole Genome Oligo Microarray (Agilent Technologies) using an incomplete loop design. Using two arrays, two *hg*/*hg *samples (one labeled with Cy3 and the other Cy5) were hybridized with two reciprocally labeled *cast/cast *samples (i.e. *hg*/*hg *Cy3 was hybridized along with *cast*/*cast *Cy5). Four additional arrays were used for the other two genotype combinations (*hg*/*hg *vs. *hg*/*cast *and *cast*/*cast *vs. *hg*/*cast*). TIFF images were obtained using the Agilent DNA Microarray Scanner BA (Agilent Technologies) and intensities were measured with the accompanying Feature Extraction Software. Intensities were normalized by linear and LOWESS regressions and background corrected by subtracting the trend of minimum intensities along the surface of the slide. Data were then analyzed with the *maanova *package [33-35] from the Bioconductor suite for R [36]. Genes were tested both for additive and dominance effects by setting the appropriate contrasts in the mixed model y_ijkl _= Dye_i _+ Array_j _+ Genotype_k _+ Error_ijkl _where Dye and Genotype are fixed and Array and Error are random effects. P-values were corrected for multiple comparisons using an FDR transformation [37].

### Expression QTL analysis

Duplicate qPCR reactions were carried out with 25 ng of cDNA using ABI Gene Expression Assays (Applied Biosystems) for each gene on the Applied Biosystems 7500 Fast Real-Time PCR System (Applied Biosystems). The ABI Gene Expression Assays were as follows: *AI451465*, Mm00505280_m1; *Fmn*, Mm00439021_m1; *Scg5*, Mm00486077_m1; *Cd44*, Mm01277160_m1; *2310032D16Rik*, Mm00512140_m1; *3300001M20Rik*, Mm00558305_m1; *Pak7*, Mm00556184_m1; *Actr5*, Mm00615134_m1; *D930001I22Rik*, Mm00557752_m1; *Sdccag33l*, Mm01248119_m1, *Rab22a*, Mm00508287_m1; and *Pcsk2 *Mm00500981_m1. *Sdha *(succinate dehydrogenase complex, subunit A, flavoprotein (fp)) and *Gus *(beta glucuronidase) were used as endogenous control genes. They are not located in the congenic region (*Sdha *is on MMU13 at 70.4 Mbp and *Gus *is on MMU5 at 129.2 Mbp) and both are common, stably expressed control genes. The control corrected expression of each target gene was determined as ΔCt = Ct (target gene) - Ct ((*Sdha *Ct + *Gus *Ct)/2). These measurements (ΔCt) were then subjected to interval mapping using the "scanone" function of R/qtl [38]. Mouse age, litter size and dam's parity were used as additive covariates. The "fitqtl" function was used to estimate genetic effects and percent variance explained.

### qPCR in B62D-3 and HG mice

qPCR reactions for *Scg5 *and *Pcsk2 *were performed in B62D-3 mice as described above. The expression of each target gene was determined using the expression, 2^-ΔCt^, where ΔCt = Ct (target gene) - Ct (control gene). These values were analyzed with SAS using a linear model that included the fixed effects of genotype [39]. The expression of *Scg5 *was also measured in whole brain from five HG and B6 mice at 3 WK, 4.5 WK and 9 WK. qPCR was carried out as described above and *Sdha *normalized expression levels (2^-ΔCt) ^were calculated for each sample. These values were analyzed with SAS using a linear model that included the fixed effects of strain, age and strain by age interaction [39]. The mean values for HG at each age were then scaled to B6 expression levels.

### Scg5 promoter sequencing

PCR amplicons (Additional File 5) covering approximately 2500 bp upstream of the *Scg5 *transcriptional start site were sequenced from the B6, HG, CAST, BALB, DBA, and C3H strains. PCR amplified fragments were gel purified using the Qiagen QIAquick gel extraction kit and sequenced at the High-Throughput Genomics Unit at the University of Washington.

### PCSK2 Enzyme Assays

All of the following extraction steps were performed at 4°C. Pituitaries were briefly sonicated in 100 mM sodium acetate, pH 5.0, and 1% Triton ×-100 in the presence of a protease inhibitor cocktail composed of 1 μM pepstatin, 1 μM *trans*-epoxysuccinic acid (E-64), and 1 mM phenylmethanesulfonyl fluoride (PMSF). The extracts were centrifuged for 2 min at 15,000 × *g*. The supernatants were used for PC2 enzyme assays. The assay for PC2 was carried out in triplicate in 96 well polypropylene microtiter plates using 10 μl of each sample in a total volume of 50 μl containing 200 μM fluorogenic substrate, pyr-Glu-Arg-Thr-Lys-Arg-methylcoumarinamide (MCA) as a substrate and 100 mM sodium acetate buffer (pH 5.0) containing 5 mM CaCl_2_, 0.5% Triton ×-100 in the presence of a protease inhibitor cocktail above with the addition of 0.14 mM tosyllysyl chloromethyl ketone (TLCK) [40]. The activity was also separately measured in the presence of 1 μM 7B2 CT peptide, a specific inhibitor of PC2 [40]. The fluorescent product MCA was measured with a Fluoroscan Ascent plate fluorometer. The amount of released product was calculated by reference to the fluorescence of the free MCA standard and is given in fluorescence units (FU) per minute, in which one FU corresponds to 5.33 pmol of MCA. These values were analyzed with SAS using a linear model that included the fixed effects of genotype [39].

### 7B2 Radioimmunoassays

Pituitaries were homogenized by sonication in 250 μl of ice-cold 0.1 N HCl. The samples were stored frozen, thawed, and centrifuged for 15 min at 13,000 rpm (17,383 × *g*) at 4°C. The supernatant was lyophilized and resuspended in 0.5 ml of radioimmunoassay (RIA) buffer (100 mM sodium phosphate, pH 7.4, containing 0.1% heat-treated BSA, 50 mM NaCl, and 0.1% sodium azide). All samples were stored frozen at -70°C until use. For 7B2 assays, 50 μl of pituitary samples were subjected to assay in duplicate. The polyclonal antiserum against 7B2 (LSU13BF), directed against residues 23–39 of 7B2 [30], was used to detect 7B2. ^125^I-labeled 7B2 was prepared by the chloramine-T method originally described by Hunter and Greenwood [41] RIAs were carried out according to protocols described previously [42]. Samples were incubated with 10,000 cpm of iodinated peptide and the appropriate dilution of rabbit antiserum in a final volume of 300 μl at 4°C overnight. To separate the antibody-bound labeled peptide from the unbound labeled peptide, 1 ml of 25% polyethylene glycol and 100 μl of 7.5% carrier bovine γ-globulin (in PBS) were added. The samples were vortexed, kept on ice for 30 min, and then centrifuged for 20 min at 3,000 × g at 4°C using a Sorvall RT6000B refrigerated centrifuge. The supernatant was aspirated, and the radioactivity in the pellets was determined. These values were analyzed with SAS using a linear model that included the fixed effects of genotype [39].

### α-MSH and ACTH assays

10 μl of a 1/200 dilution pituitary sample in RIA buffer were subjected to assay in duplicate. The polyclonal anti-α-MSH antiserum was commercially purchased from Chemicon (Temecula, CA). α-MSH assays were performed as described above. ACTH assays were carried out using the two-site Nichols human ACTH_1–39 _assay kit (Nichols Institute, San Juan Capistrano, CA). The ^125^I-ACTH antibody used in this kit is directed towards both N-terminal and C-terminal regions of intact ACTH molecule and does not recognize ACTH cleavage products. These values were analyzed with SAS using a linear model that included the fixed effects of genotype [39].

## Authors' contributions

CRF, IL and JFM conceived the study. CRF, JC, SNL, RAV and AIT performed all experiments. CRF, RAV and JFM analyzed the data. CRF, IL and JFM drafted the manuscript. JFM provided coordination of the project. All authors read and approved the final manuscript.

## Supplementary Material

Additional file 1 Differentially expressed genes in HG2DF2 non-recombinant F2 mice. These data represent a list of genes differentially expressed in HG2DF2 non-recombinant F2 mice determined using DNA microarrays. Click here for file

Additional file 2 *Scg5 *whole brain expression is up-regulated in HG versus B6 male mice at 3, 4.5 and 9 weeks of age and the *Scg5 *promoter contains two tandem putative *Stat5b *DNA binding sites. Expression analysis of *Scg5 *as a function of HG genotype. Click here for file

Additional file 3 *Scg5 *haplotypes based on available SNP data. Haplotype analysis of *Scg5 *in "low" and "high" expressing strains using publically available SNP data. Click here for file

Additional file 4 Hierarchical clustering analysis of *Scg5 *haplotype blocks. Illustrates the relationships of *Scg5 *haplotype blocks in "low" and "high" expressing strains. Click here for file

Additional file 5 *Scg5 *promoter sequencing primers. List of PCR primers used for *Scg5 *sequencing. Click here for file
