# Supplementary material for: Overexpression of Scg5 increases enzymatic activity of PCSK2 and is inversely correlated with body weight in congenic mice
Source: BMC Genet. 2008 Apr 25;9:34. doi: 10.1186/1471-2156-9-34 (PMC2386500; doi:10.1186/1471-2156-9-34)
Supplement: Additional file 1 — Differentially expressed genes in HG2DF2 non-recombinant F2 mice. These data represent a list of genes differentially expressed in HG2DF2 non-recombinant F2 mice determined using DNA microarrays. [file 1471-2156-9-34-S1.pdf]

ADDITIONAL FILE 1. Differentially expressed genes in HG2DF2 non-recombinant F2 mice

Gene Name	Agilent Probe ID	Accession	FDR	Chr.	Mbp UCSC Feb. 2006	Fold change	High expressing strain
Drbp1	A_51_P451374	NM_153405	0.222	2	76.171	6.09	CAST
AK078845	A_52_P1052731	AK078845	0.102	2	78.685	7.69	HG
D430039N05Rik	A_51_P393654	NM_175514	0.291	2	83.614	2.30	HG
Olfr1153	A_52_P361391	NM_146640	0.113	2	87.697	2.73	HG
AA718294	A_51_P308111	AA718294	0.220	2	90.720	1.93	HG
Slc39a13	A_52_P6505	AK035296	0.220	2	90.863	1.76	CAST
E130006N16Rik	A_51_P139765	AK053282	0.236	2	91.082	2.70	HG
2810427C15Rik	A_52_P761812	AK013171	0.121	2	92.107	6.46	HG
D030065N23Rik	A_52_P1116376	AK083682	0.266	2	92.132	3.42	HG
2810002D19Rik	A_52_P158305	BC022909	0.256	2	94.207	2.26	HG
Cd44	A_52_P229536	AK045226	0.150	2	102.515	1.81	HG
AK051363	A_52_P671465	AK051363	0.278	2	103.589	1.66	HG
AK031920	A_52_P803354	AK031920	0.182	2	104.439	3.46	HG
AI451465	A_51_P204286	AK033552	0.266	2	112.272	1.66	HG
Fmn1	A_52_P321725	X62379	0.097	2	113.242	6.46	CAST
Scg5	A_51_P221223	NM_009162	0.102	2	113.578	2.96	CAST
Ivd	A_51_P425680	NM_019826	0.182	2	118.553	1.44	HG
Mga	A_51_P193270	NM_013720	0.266	2	119.564	2.07	HG
2310026J01Rik	A_52_P203948	BC066085	0.216	2	119.858	4.97	CAST
Vps39	A_51_P460828	BC007479	0.097	2	120.008	2.96	CAST
5830445O15Rik	A_52_P663742	AK076333	0.220	2	120.098	1.41	CAST
4831403C07Rik	A_51_P376501	AK029170	0.219	2	120.396	1.35	CAST
B430315C20Rik	A_52_P529790	NM_178795	0.102	2	121.002	6.03	HG
Sema6d	A_51_P415755	AK052232	0.121	2	124.302	2.59	CAST
Znf2	A_51_P105869	NM_028141	0.278	2	127.267	2.30	HG
Mal	A_52_P562661	NM_010762	0.172	2	127.325	1.97	HG
Stk35	A_52_P451834	AK085193	0.097	2	129.492	2.62	HG
Gfra4	A_51_P241426	NM_020014	0.287	2	130.732	4.11	HG
2310032D16Rik	A_52_P470017	NM_028802	0.102	2	132.221	5.78	HG
3300001M20Rik	A_52_P525837	NM_175113	0.220	2	132.496	2.53	HG
D2Bwg1356e	A_51_P319379	AK129301	0.287	2	134.286	1.19	HG
AK086068	A_52_P739620	AK086068	0.219	2	134.935	3.42	HG

Gene Name	Agilent Probe ID	Accession	FDR	Chr.	Mbp UCSC Feb. 2006	Fold change	High expressing strain
Pak7	A_52_P143252	AK077967	0.121	2	135.773	3.03	HG
Snap25	A_52_P286421	NM_011428	0.102	2	136.405	8.71	HG
AK045769	A_52_P1027959	AK045769	0.185	2	140.524	5.03	HG
AK028486	A_52_P763142	AK028486	0.097	2	140.787	27.23	HG
1700006F03Rik	A_51_P131840	AF440737	0.097	2	148.481	19.12	CAST
Cst3	A_51_P332201	NM_009976	0.278	2	148.563	1.72	CAST
BC029716	A_52_P471592	BC034102	0.097	2	149.695	3.38	CAST
AL033326	A_51_P233578	NM_019705	0.102	2	152.008	11.88	HG
H13	A_52_P655663	NM_010376	0.278	2	152.361	1.84	CAST
Pofut1	A_52_P665833	NM_080463	0.172	2	152.933	2.24	HG
9830001H06Rik	A_52_P257919	AK031493	0.222	2	156.707	2.25	HG
2610304G08Rik	A_51_P229633	NM_027434	0.230	2	157.720	3.06	HG
Actr5	A_52_P594049	NM_175419	0.182	2	158.316	1.86	HG
D930001I22Rik	A_52_P172441	NM_173397	0.256	2	163.158	1.33	CAST
AK045608	A_52_P1067686	AK045608	0.216	2	164.183	2.15	HG
AK038166	A_51_P389147	AK038166	0.232	2	165.842	6.42	HG
Sdccag33l	A_52_P126663	AK046877	0.172	2	169.325	2.19	CAST
2900073F20Rik	A_52_P158431	AK013774	0.220	2	169.331	3.00	CAST
Rab22a	A_52_P582767	NM_024436	0.102	2	173.303	2.55	HG
2210418O10Rik	A_51_P206849	BC059210	0.219	2	176.503	1.88	CAST
LOC381422	A_52_P347796	XM_358573	0.186	2	176.899	2.32	CAST
BC025828	A_52_P310652	BC025828	0.182	2	177.478	2.89	CAST
C330013J21Rik	A_51_P211423	AK021199	0.102	2	177.833	2.18	CAST
Genes outside HG2D congenic region							
BB755555	A_52_P708886	BB755555	0.182	1	7.169	2.18	HG
Prg4	A_51_P280455	AB034730	0.219	1	152.214	1.38	HG
Oas2	A_51_P277994	NM_145227	0.182	5	120.991	1.56	CAST
9330160C06Rik	A_51_P481528	AK034156	0.182	6	149.056	1.17	HG
6230410P16Rik	A_51_P194004	NM_174846	0.172	9	106.011	2.16	HG
Igtp	A_51_P112355	NM_018738	0.097	11	58.0158	1.29	CAST
Egr1	A_51_P367866	NM_007913	0.133	18	34.987	1.31	CAST
