# Supplementary material for: Overexpression of Scg5 increases enzymatic activity of PCSK2 and is inversely correlated with body weight in congenic mice
Source: BMC Genet. 2008 Apr 25;9:34. doi: 10.1186/1471-2156-9-34 (PMC2386500; doi:10.1186/1471-2156-9-34)

ADDITIONAL FILE 2: *Sgne1* whole brain expression is upregulated in HG versus B6 male mice at 3, 4.5 and 9 weeks of age and the *Sgne1* promoter contains two tandem putative *Stat5b* DNA binding sites. A) Relative *Sgne1* expression is increased approximately 25% at 3 weeks and 40% at 4.5 weeks and 9 weeks of age. N = 5 for both strains at all ages. *Sgne1* expression at each age was scaled to levels of expression in B6 mice. B) *In silico* analysis revealed six *Stat5b* DNA binding sites (TTCYNRGAA) within the *Sgne1* gene. *Sgne1* exons are signified by black boxes and the position of *Stat5b* sites are labeled with a (★). Two of the sites were in tandem and located between -56 and -39 bp upstream of the transcription start site.

A

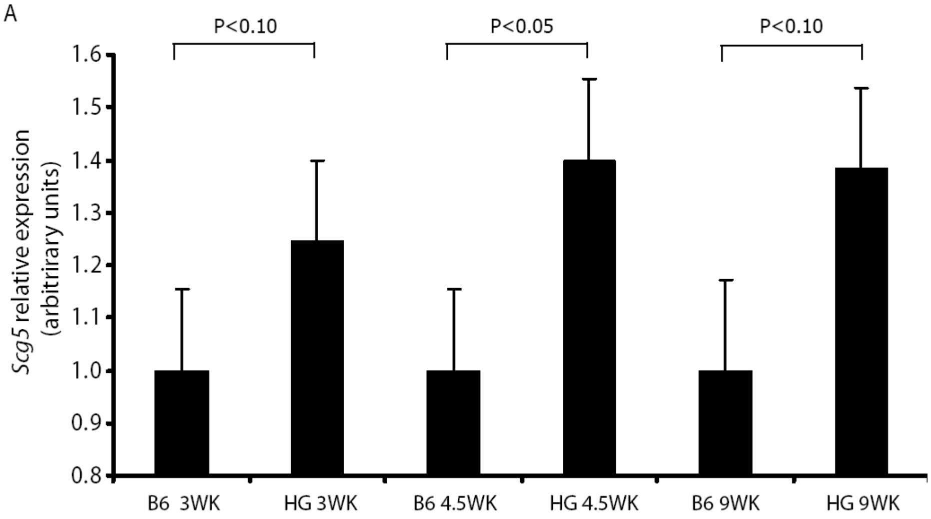

B

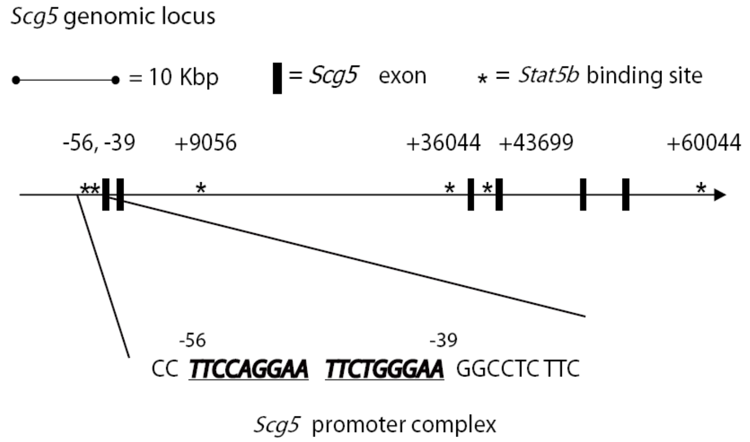

Supplement: Additional file 2 — Scg5 whole brain expression is up-regulated in HG versus B6 male mice at 3, 4.5 and 9 weeks of age and the Scg5 promoter contains two tandem putative Stat5b DNA binding sites. Expression analysis of Scg5 as a function of HG genotype. [file 1471-2156-9-34-S2.pdf]
