# Supplementary material for: Overexpression of Scg5 increases enzymatic activity of PCSK2 and is inversely correlated with body weight in congenic mice
Source: BMC Genet. 2008 Apr 25;9:34. doi: 10.1186/1471-2156-9-34 (PMC2386500; doi:10.1186/1471-2156-9-34)
Supplement: Additional file 3 — Scg5 haplotypes based on available SNP data. Haplotype analysis of Scg5 in "low" and "high" expressing strains using publically available SNP data. [file 1471-2156-9-34-S3.pdf]

ADDITIONAL FILE 3. *Scg5* haplotypes based on availabe SNP data. Haplotype blocks were determined using the haploview software.
Haplotype blocks are shaded, block 1 in grey and block 2 in dark green. SNPs labeled "YES" are candidates based on the critieria of being unique to B6. Mbp location determined from NCBI mouse genome build 36. SNPs data downloaded from Mouse Phenome Database (http://www.jax.org/phenome)

Mbp location	NCBI gene annotation	Ensembl gene annotation	dbSNP SNP annotation	C57BL/6J	C3H/HeJ	BALB/cByJ	DBA/2J	CAST/EiJ	Haplotype Candidate (YES)	dbSNP rs	Observed	Source
02 113.571079				G	T			G		rs27491143	G/T	Perlegen2 NES09039595
02 113.571212				A	G			A		rs27491142	A/G	Perlegen2 NES09039596
02 113.571276				T	A			T		rs27491141	A/T	Perlegen2 NES09039597
02 113.571368				T	C		C	C		rs27491140	C/T	multi multi
02 113.571411				G	A			G		rs27491139	A/G	Perlegen2 NES09039599
02 113.571450				A	T			A		rs27491138	A/T	Perlegen2 NES09039600
02 113.571674				T	A			T		rs27491137	A/T	Perlegen2 NES09039601
02 113.571733				T	A			T		rs27491136	A/T	Perlegen2 NES09039602
02 113.571753				T	C			T		rs27491135	C/T	Perlegen2 NES09039603
02 113.571785				T	T			C		rs27491134	C/T	Perlegen2 NES09039604
02 113.571862				G	G			A		rs27491133	A/G	Perlegen2 NES09039605
02 113.571914				C	C			C		rs27491132	A/C	Perlegen2 NES09039606
02 113.572027				T	C			T		rs27491131	C/T	Perlegen2 NES09039607
02 113.572235				C	T			C		rs27491130	C/T	Perlegen2 NES09039608
02 113.572734				A	T			A		rs27491129	A/T	Perlegen2 NES09039483
02 113.572825				C	C			C		rs27491128	C/T	Perlegen2 NES09039484
02 113.572852				C	T			C		rs27491127	C/T	Perlegen2 NES09039485
02 113.572899				A	G			A		rs27491126	A/G	Perlegen2 NES09039487
02 113.572970				G	A			G		rs27491125	A/G	Perlegen2 NES09039489
02 113.572985				T	C			T		rs27491124	C/T	Perlegen2 NES09039490
02 113.573795				G	C			G		rs27491123	C/G	Perlegen2 NES09039451
02 113.573961				G	A			G		rs27491122	A/G	Perlegen2 NES09039452
02 113.574251				A	G		G	A		rs27491121	A/G	multi multi
02 113.574473				T	C			C		rs27491120	C/T	Perlegen2 NES09039454
02 113.574658				G	T			G		rs27491119	G/T	Perlegen2 NES09039418
02 113.574757				G	G			G		rs27491118	A/G	Perlegen2 NES09039420
02 113.574866				A	G			G		rs27491117	A/G	Perlegen2 NES09039422
02 113.575254				G	A			G		rs27491116	A/G	Perlegen2 NES09039423
02 113.575425				C	T			T		rs27491115	C/T	Perlegen2 NES09039424
02 113.575494				C	T			T		rs27491114	C/T	Perlegen2 NES09039425
02 113.575600				A	G			A		rs27491113	A/G	Perlegen2 NES09039426
02 113.575788				C	G			C		rs27491112	C/G	Perlegen2 NES09039427
02 113.575905				G	G			A		rs27491111	A/G	Perlegen2 NES09039428
02 113.575909				G	T			G		rs27491110	G/T	Perlegen2 NES09039429
02 113.576004				C	G			C		rs27491109	C/G	Perlegen2 NES09039430
02 113.576018				C	T			C		rs27491108	C/T	Perlegen2 NES09039431
02 113.576267				A	C		C	C		rs27491107	A/C	multi multi
02 113.576506				C	C			C		rs27491106	C/T	Perlegen2 NES09039433

Mbp location	NCBI gene annotation	Ensembl gene annotation	dbSNP SNP annotation	C57BL/6J	C3H/HeJ	BALB/cByJ	DBA/2J	CAST/EiJ	Haplotype Candidate (YES)	dbSNP rs	Observed	Source
02 113.577084			Scg5 L	T	C					rs27491105	C/T	Perlegen2 NES09039290
02 113.577782	Scg5_intron5	agrees	agrees	G	A	G	G	G		rs27491104	A/G	Perlegen2 NES09039291
02 113.577898	Scg5_intron5	agrees	agrees	G	G	G	G	A		rs27491103	A/G	Perlegen2 NES09039292
02 113.578363	Scg5_intron5	agrees	agrees	C	C	T	T	C		rs27491102	C/T	multi multi
02 113.578369	Scg5_intron5	agrees	agrees	A	G		A	A		rs27491101	A/G	Perlegen2 NES09039295
02 113.578494	Scg5_intron5	agrees	agrees	T	C	C	C	T		rs27491100	C/T	multi multi
02 113.578630	Scg5_intron5	agrees	agrees	A	C	C	C			rs27491099	A/C	Perlegen2 NES09039299
02 113.578944	Scg5_intron5	agrees	agrees	G	G	C	C	G		rs27491098	C/G	multi multi
02 113.578960	Scg5_intron5	agrees	agrees	C	A	C	C	C		rs27491097	A/C	Perlegen2 NES09039302
02 113.579646	Scg5_intron5	agrees	agrees	T	C	T	T	T		rs27491096	C/T	Perlegen2 NES09039254
02 113.579890	Scg5_intron5	agrees	agrees	A	G	A	A	A		rs27491095	A/G	Perlegen2 NES09039255
02 113.580098	Scg5_intron5	agrees	agrees	T	C	C	C	C	YES	rs27491094	C/T	multi multi
02 113.580371	Scg5_intron5	agrees	agrees	C	C	C	C	T		rs27491093	C/T	Perlegen2 NES09039257
02 113.580727	Scg5_intron5	agrees	agrees	T	C	T	T	T		rs27491092	C/T	Perlegen2 NES09039258
02 113.580881	Scg5_intron5	agrees	agrees	T	G	T	T	T		rs27491091	G/T	Perlegen2 NES09039259
02 113.580917	Scg5_intron5	agrees	agrees	A	G	A	A	A		rs27491090	A/G	Perlegen2 NES09039260
02 113.581344	Scg5_intron5	agrees	agrees	C	G	C	C	C		rs27491089	C/G	Perlegen2 NES09039261
02 113.581379	Scg5_intron5	agrees	agrees	A	A	C	C	A		rs27491088	A/C	multi multi
02 113.581454	Scg5_intron5	agrees	agrees	A	G	A	A	A		rs27491087	A/G	Perlegen2 NES09039263
02 113.581710	Scg5_intron4	agrees	agrees	C	T	T	T	C		rs27491086	C/T	multi multi
02 113.581772	Scg5_intron4	agrees	agrees	G	A	A	A	A	YES	rs27491085	A/G	multi multi
02 113.581814	Scg5_intron4	agrees	agrees	T	C	T	T	T		rs27491084	C/T	Perlegen2 NES09039266
02 113.581959	Scg5_intron4	agrees	agrees	A	G	A	A	A		rs27491083	A/G	Perlegen2 NES09039267
02 113.581978	Scg5_intron4	agrees	agrees	C	C	T	T	C		rs27491082	C/T	multi multi
02 113.582212	Scg5_intron4	agrees	agrees	G	G	A	A	A		rs27491081	A/G	Perlegen2 NES09039272
02 113.582642	Scg5_intron4	agrees	agrees	T	T	T	T	T		rs27491080	C/T	Perlegen2 NES09039274
02 113.582743	Scg5_intron4	agrees	agrees	A	G	A	A	A		rs27491079	A/G	Perlegen2 NES09039276
02 113.583014	Scg5_intron4	agrees	agrees	G	G	G	G	A		rs27491078	A/G	Perlegen2 NES09039278
02 113.585121	Scg5_intron4	agrees	agrees	C	C	C	C	C		rs27491077	C/T	Perlegen2 NES09039217
02 113.585243	Scg5_intron4	agrees		G	A	A	A	G				Perlegen2 NES09039218
02 113.588130	Scg5_intron4	agrees	agrees	C	C	C	C	C		rs27491075	A/C	Perlegen2 NES09039140
02 113.589240	Scg5_intron4	agrees	agrees	T	T	T	T	T		rs27491074	A/T	Perlegen2 NES09039131
02 113.589442	Scg5_intron4	agrees	agrees	G	G	G	G	G		rs27491073	A/G	Perlegen2 NES09039132
02 113.589665	Scg5_exon4	agrees	**Cs**	G	G	G	G	A		rs27491072	A/G	Perlegen2 NES09039133
02 113.589798	Scg5_intron3	agrees	agrees	T	T	T	T	T		rs27491071	G/T	Perlegen2 NES09039134
02 113.589852	Scg5_intron3	agrees	agrees	A	A	A	A	A		rs27491070	A/G	Perlegen2 NES09039135
02 113.590439	Scg5_intron3	agrees	agrees	G	G	G	G	G		rs27491069	A/G	Perlegen2 NES09039111
02 113.590536	Scg5_intron3	agrees	agrees	T	T	T	T	T		rs27491068	C/T	Perlegen2 NES09039113
02 113.591337	Scg5_intron3	agrees	agrees	A	A	A	A	A		rs27491067	A/G	Perlegen2 NES09039082
02 113.591876	Scg5_intron3	agrees	agrees	T	T	T	T	T		rs27491066	A/T	Perlegen2 NES09039083
02 113.591981	Scg5_intron3	agrees	agrees	C	C	C	C	C		rs27491065	C/T	Perlegen2 NES09039084
02 113.592438	Scg5_intron3	agrees	agrees	C	C	C	C	C		rs27491064	A/C	Perlegen2 NES09039072

Mbp location	NCBI gene annotation	Ensembl gene annotation	dbSNP SNP annotation	C57BL/6J	C3H/HeJ	BALB/cByJ	DBA/2J	CAST/EiJ	Haplotype Candidate (YES)	dbSNP rs	Observed	Source
02 113.592957	Scg5 intron2	agrees	agrees	G	A	A	A			rs27491063	A/G	multi multi
02 113.592961	Scg5 intron2	agrees	agrees	C	C	C	C	T		rs27491062	C/T	Perlegen2 NES09039074
02 113.593406	Scg5 intron2	agrees	agrees	A	G	G	G	A		rs27491061	A/G	multi multi
02 113.593702	Scg5 intron2	agrees	agrees	G	G	G	G			rs27491060	A/G	Perlegen2 NES09039028
02 113.594205	Scg5 intron2	agrees	agrees	T	T	T	T	T		rs27491059	A/T	Perlegen2 NES09038999
02 113.594399	Scg5 intron2	agrees	agrees	C	C	C	C			rs27491058	C/T	Perlegen2 NES09039001
02 113.594520	Scg5 intron2	agrees	agrees	T	C	C	C	C	YES	rs27491057	C/T	multi multi
02 113.594636	Scg5 intron2	agrees	agrees	A	A	A	A	A		rs27491056	A/G	Perlegen2 NES09039006
02 113.594736	Scg5 intron2	agrees	agrees	T	T	T	T	T		rs27491055	G/T	Perlegen2 NES09039009
02 113.595207	Scg5 intron2	agrees	agrees	A	A	A	A	A		rs27491054	A/C	Perlegen2 NES09039011
02 113.596123	Scg5 intron2	agrees	agrees	T	T	T	T	T		rs27491053	C/T	Perlegen2 NES09038943
02 113.596542	Scg5 intron2	agrees	agrees	G	G	G	G	G		rs27491052	A/G	Perlegen2 NES09038944
02 113.596685	Scg5 intron2	agrees	agrees	T	T	T	T	T		rs27491051	C/T	Perlegen2 NES09038945
02 113.597539	Scg5 intron2	agrees	agrees	A	A		A	A		rs27491050	A/G	Perlegen2 NES09038913
02 113.597626	Scg5 intron2	agrees	agrees	C	C	C	C	C		rs27491049	C/G	Perlegen2 NES09038914
02 113.597707	Scg5 intron2	agrees	agrees	A	A	A	A	A		rs27491048	A/G	Perlegen2 NES09038915
02 113.597833	Scg5 intron2	agrees	agrees	T	T	T	T	T		rs27491047	C/T	Perlegen2 NES09038916
02 113.598190	Scg5 intron2	agrees	agrees	G	G	G	G	G		rs27491046	A/G	Perlegen2 NES09038898
02 113.598428	Scg5 intron2	agrees	agrees	T	T	T	T	T		rs27491045	C/T	Perlegen2 NES09038869
02 113.598593	Scg5 intron2	agrees	agrees	A	A	A	A	A		rs27491044	A/G	Perlegen2 NES09038872
02 113.598832	Scg5 intron2	agrees	agrees	T	G	G	G	G	YES	rs27491043	G/T	Perlegen2 NES09038875
02 113.598870	Scg5 intron2	agrees	agrees	A	A	A	A	A		rs27491042	A/C	Perlegen2 NES09038877
02 113.599828	Scg5 intron2	agrees	agrees	G	G	G	G	G		rs27491041	C/G	Perlegen2 NES09038879
02 113.600015	Scg5 intron2	agrees	agrees	T	T	T	T	T		rs27491040	G/T	Perlegen2 NES09038881
02 113.600213	Scg5 intron2	agrees	agrees	G	G	G	G	G		rs27491039	A/G	Perlegen2 NES09038883
02 113.600233	Scg5 intron2	agrees	agrees	C	C	C	C	C		rs27491038	A/C	Perlegen2 NES09038885
02 113.600730	Scg5 intron2	agrees	agrees	G	G	G	G	G		rs27491037	A/G	Perlegen2 NES09038840
02 113.600814	Scg5 intron2	agrees	agrees	A	A	A	A	A		rs27491036	A/G	Perlegen2 NES09038841
02 113.600989	Scg5 intron2	agrees	agrees	C	C	C	C	C		rs27491035	C/T	Perlegen2 NES09038805
02 113.601281	Scg5 intron2	agrees	agrees	G	G	G	G	G		rs27491034	A/G	Perlegen2 NES09038806
02 113.601305	Scg5 intron2	agrees	agrees	G	G	G	G	G		rs27491033	C/G	Perlegen2 NES09038807
02 113.601389	Scg5 intron2	agrees	agrees	T	T	T	T	T		rs27491032	G/T	Perlegen2 NES09038808
02 113.601472	Scg5 intron2	agrees	agrees	T	T	T	T	T		rs27491031	C/T	Perlegen2 NES09038809
02 113.601484	Scg5 intron2	agrees	agrees	G	G	G	G	G		rs27491030	A/G	Perlegen2 NES09038810
02 113.601542	Scg5 intron2	agrees	agrees	A	A	A	A	A		rs27491029	A/G	Perlegen2 NES09038811
02 113.601654	Scg5 intron2	agrees	agrees	C	C	C	C	C		rs27491028	C/T	Perlegen2 NES09038812
02 113.601669	Scg5 intron2	agrees	agrees	C	C	C	C	C		rs27491027	C/T	Perlegen2 NES09038813
02 113.601879	Scg5 intron2	agrees	agrees	C	C	C	C	C		rs27491026	C/G	Perlegen2 NES09038814
02 113.602179	Scg5 intron2	agrees	agrees	G	A	A	A	A	YES	rs27491025	A/G	multi multi
02 113.602219	Scg5 intron2	agrees	agrees	A	A	A	A	A		rs27491024	A/G	Perlegen2 NES09038818
02 113.602414	Scg5 intron2	agrees	agrees	C	C	C	C	C		rs27491023	A/C	Perlegen2 NES09038819
02 113.602424	Scg5 intron2	agrees	agrees	T	T	T	T	T		rs27491022	A/T	Perlegen2 NES09038820

Mbp location	NCBI gene annotation	Ensembl gene annotation	dbSNP SNP annotation	C57BL/6J	C3H/HeJ	BALB/cByJ	DBA/2J	CAST/EiJ	Haplotype Candidate (YES)	dbSNP rs	Observed	Source
02 113.602635	Scg5_intron2	agrees	agrees	C	C	C	C	C		rs27491021	C/G	Perlegen2 NES09038821
02 113.602832	Scg5_intron2	agrees	agrees	T	T	T	T	T		rs27491020	C/T	Perlegen2 NES09038822
02 113.602879	Scg5_intron2	agrees	agrees	A	A	A	A	A		rs27491019	A/C	Perlegen2 NES09038823
02 113.602892	Scg5_intron2	agrees	agrees	C	C	C	C	T		rs27491018	C/T	Perlegen2 NES09038824
02 113.603255	Scg5_intron2	agrees	agrees	A	A	A	A	A		rs27491017	A/C	Perlegen2 NES09038825
02 113.603369	Scg5_intron2	agrees	agrees	A	A	A	A	A		rs27491016	A/G	Perlegen2 NES09038826
02 113.603426	Scg5_intron2	agrees	agrees	G	G	G	G	G		rs27491015	C/G	Perlegen2 NES09038827
02 113.603623	Scg5_intron2	agrees	agrees	G	G	G	G	G		rs27491014	C/G	Perlegen2 NES09038828
02 113.603990	Scg5_intron2	agrees	agrees	T	T	T	T	T		rs27491013	A/T	Perlegen2 NES09038758
02 113.604016	Scg5_intron2	agrees	agrees	C	C	C	C	C		rs27491012	C/T	Perlegen2 NES09038759
02 113.604177	Scg5_intron2	agrees	agrees	G	G	G	G	G		rs27491011	A/G	Perlegen2 NES09038760
02 113.604217	Scg5_intron2	agrees	agrees	A	A	A	A	A		rs27491010	A/T	Perlegen2 NES09038761
02 113.604284	Scg5_intron2	agrees	agrees	A	A	A	A	A		rs27491009	A/C	Perlegen2 NES09038762
02 113.604354	Scg5_intron2	agrees	agrees	C	C	C	C	C		rs27491008	C/T	Perlegen2 NES09038763
02 113.605103	Scg5_intron2	agrees	agrees	T	C	C	C	C	YES	rs27491007	C/T	Perlegen2 NES09038744
02 113.605614	Scg5_intron2	agrees	agrees	T	G	G	G	G	YES	rs27491006	G/T	Perlegen2 NES09038702
02 113.605719	Scg5_intron2	agrees	agrees	A	T	T	T	T	YES	rs27491005	A/T	Perlegen2 NES09038703
02 113.605777	Scg5_intron2	agrees	agrees	C	C	C	C	C		rs27491004	C/G	Perlegen2 NES09038704
02 113.605802	Scg5_intron2	agrees	agrees	T						rs27491003	C/T	Perlegen2 NES09038705
02 113.605813	Scg5_intron2	agrees	agrees	A						rs27491002	A/G	Perlegen2 NES09038706
02 113.605881	Scg5_intron2	agrees	agrees	G	G	G	G	G		rs27491001	A/G	Perlegen2 NES09038707
02 113.605923	Scg5_intron2	agrees	agrees	G	A	A	A	A	YES	rs27491000	A/G	Perlegen2 NES09038708
02 113.606162	Scg5_intron2	agrees	agrees	A	A	A	A	A		rs27490999	A/C	Perlegen2 NES09038709
02 113.606174	Scg5_intron2	agrees	agrees	T	C	C	C	C	YES	rs27490998	C/T	Perlegen2 NES09038710
02 113.606197	Scg5_intron2	agrees	agrees	T	C	C	C	C	YES	rs27490997	C/T	Perlegen2 NES09038711
02 113.606226	Scg5_intron2	agrees	agrees	T	T	T	T	T		rs27490996	C/T	Perlegen2 NES09038712
02 113.606458	Scg5_intron2	agrees	agrees	A	A	A	A	A		rs27490995	A/C	Perlegen2 NES09038713
02 113.606576	Scg5_intron2	agrees	agrees	G	G	G	G	G		rs27490994	A/G	Perlegen2 NES09038714
02 113.606614	Scg5_intron2	agrees	agrees	T	T	T	T	T		rs27490993	C/T	Perlegen2 NES09038715
02 113.607345	Scg5_intron2	agrees	agrees	T	T	T	T	T		rs27490992	G/T	Perlegen2 NES09038647
02 113.607567	Scg5_intron2	agrees	agrees	G	T	T	T	G		rs27490991	G/T	multi multi
02 113.607625	Scg5_intron2	agrees	agrees	G	G	G	G	G		rs27490990	A/G	Perlegen2 NES09038649
02 113.607663	Scg5_intron2	agrees	agrees	G	G	G	G	G		rs27490989	A/G	Perlegen2 NES09038650
02 113.608553	Scg5_intron2	agrees	agrees	G	G	G	G	G		rs27490988	G/T	Perlegen2 NES09038634
02 113.608601	Scg5_intron2	agrees	agrees	A	A	A	A	A		rs27490987	A/G	Perlegen2 NES09038635
02 113.610408	Scg5_intron2	agrees	agrees	T	T	T	T	T		rs27490986	C/T	Perlegen2 NES09038498
02 113.610452	Scg5_intron2	agrees	agrees	T	T	T	T	T		rs27490985	C/T	Perlegen2 NES09038499
02 113.618679	Scg5_intron2	agrees	agrees	C	C	C	C	C		rs27490984	C/T	Perlegen2 NES09038464
02 113.618721	Scg5_intron2	agrees	agrees	G	A	A	A	A	YES	rs27490983	A/G	Perlegen2 NES09038465
02 113.619489	Scg5_intron2	agrees	agrees	G	G	G	G	A		rs27490982	A/G	Perlegen2 NES09038466
02 113.619566	Scg5_intron2	agrees	agrees	C	T	T	T	T	YES	rs27490981	C/T	Perlegen2 NES09038467
02 113.619728	Scg5_intron2	agrees	agrees	A	G	G	G	G	YES	rs27490980	A/G	Perlegen2 NES09038468

Mbp location	NCBI gene annotation	Ensembl gene annotation	dbSNP SNP annotation	C57BL/6J	C3H/HeJ	BALB/cByJ	DBA/2J	CAST/EiJ	Haplotype Candidate (YES)	dbSNP rs	Observed	Source
02 113.620035	Scg5_intron2	agrees	agrees	C	T	T	T	T	YES	rs27490979	C/T	Perlegen2 NES09038469
02 113.620449	Scg5_intron2	agrees	agrees	C	T	T	T	T	YES	rs27490978	C/T	Perlegen2 NES09038415
02 113.620700	Scg5_intron2	agrees	agrees	G	A	A	A	A	YES	rs27490977	A/G	multi multi
02 113.621219	Scg5_intron2	agrees	agrees	T	C	C	C	C	YES	rs27490976	C/T	multi multi
02 113.621590	Scg5_intron2	agrees	agrees	T			C			rs6321486	C/T	multi multi
02 113.621850	Scg5_intron2	agrees	agrees	C				T		rs27490975	C/T	Perlegen2 NES09038330
02 113.622096	Scg5_intron2	agrees	agrees	G				A		rs27490974	A/G	Perlegen2 NES09038331
02 113.622263	Scg5_intron2	agrees	agrees	G						rs27490973	A/G	Perlegen2 NES09038332
02 113.622479	Scg5_intron2	agrees	agrees	T						rs27490972	C/T	Perlegen2 NES09038333
02 113.623120	Scg5_intron2	agrees	agrees	C			T	T		rs27490971	C/T	multi multi
02 113.623185	Scg5_intron2	agrees	agrees	T			T			rs27490970	C/T	Perlegen2 NES09038280
02 113.624307	Scg5_intron2	agrees	agrees	G				C		rs27490969	C/G	Perlegen2 NES09038209
02 113.625966	Scg5_intron2	agrees	agrees	C						rs27490968	C/G	Perlegen2 NES09037989
02 113.626186	Scg5_intron2	agrees	agrees	C						rs27469967	A/C	Perlegen2 NES09037992
02 113.627165	Scg5_intron2	agrees	agrees	C						rs27469966	C/G	Perlegen2 NES09037997
02 113.627360	Scg5_intron2	agrees	agrees	G						rs27469965	A/G	Perlegen2 NES09037999
02 113.628017	Scg5_intron2	agrees	agrees	C	C	C	C	C		rs4223402	C/T	GNF1 X15830_19
02 113.628037	Scg5_intron2	agrees	agrees	T	T	T	T	T		rs4223403	A/T	GNF1 X15830_39
02 113.628046	Scg5_intron2	agrees	agrees	C	C	C	C	C		rs4223404	C/T	GNF1 X15830_48
02 113.628141	Scg5_intron2	agrees	agrees	T	T	T	T	C		rs4223405	C/T	GNF1 X15830_143
02 113.628191	Scg5_exon2	agrees	**Cs**	T	C	C	C	C	YES	rs4223406	C/T	multi multi
02 113.628380	Scg5_exon2	agrees	**Cs**	A	G	G	G	G	YES	rs4223407	A/G	multi multi
02 113.628388	Scg5_exon2	agrees	**Cn**	C	C	C	C	C		rs4223408	C/G	GNF1 X15830_390
02 113.628392	Scg5_exon2	agrees	**Cs**	G	G	G	G	C		rs4223409	C/G/T	GNF1 X15830_394
02 113.628894	Scg5_intron1	agrees	Scg5 UTR	A						rs27469964	A/G	Perlegen2 NES09038002
02 113.629342	Scg5_intron1	agrees		T	C		C	C				Broad1 2-113719477
02 113.633403	Arhgap11a_intron12	agrees	UTR	A				A		rs27469963	A/C	Perlegen2 NES09038006
