# Supplementary figures and images for: Overexpression of Scg5 increases enzymatic activity of PCSK2 and is inversely correlated with body weight in congenic mice
Source: BMC Genet. 2008 Apr 25;9:34. doi: 10.1186/1471-2156-9-34 (PMC2386500; doi:10.1186/1471-2156-9-34)

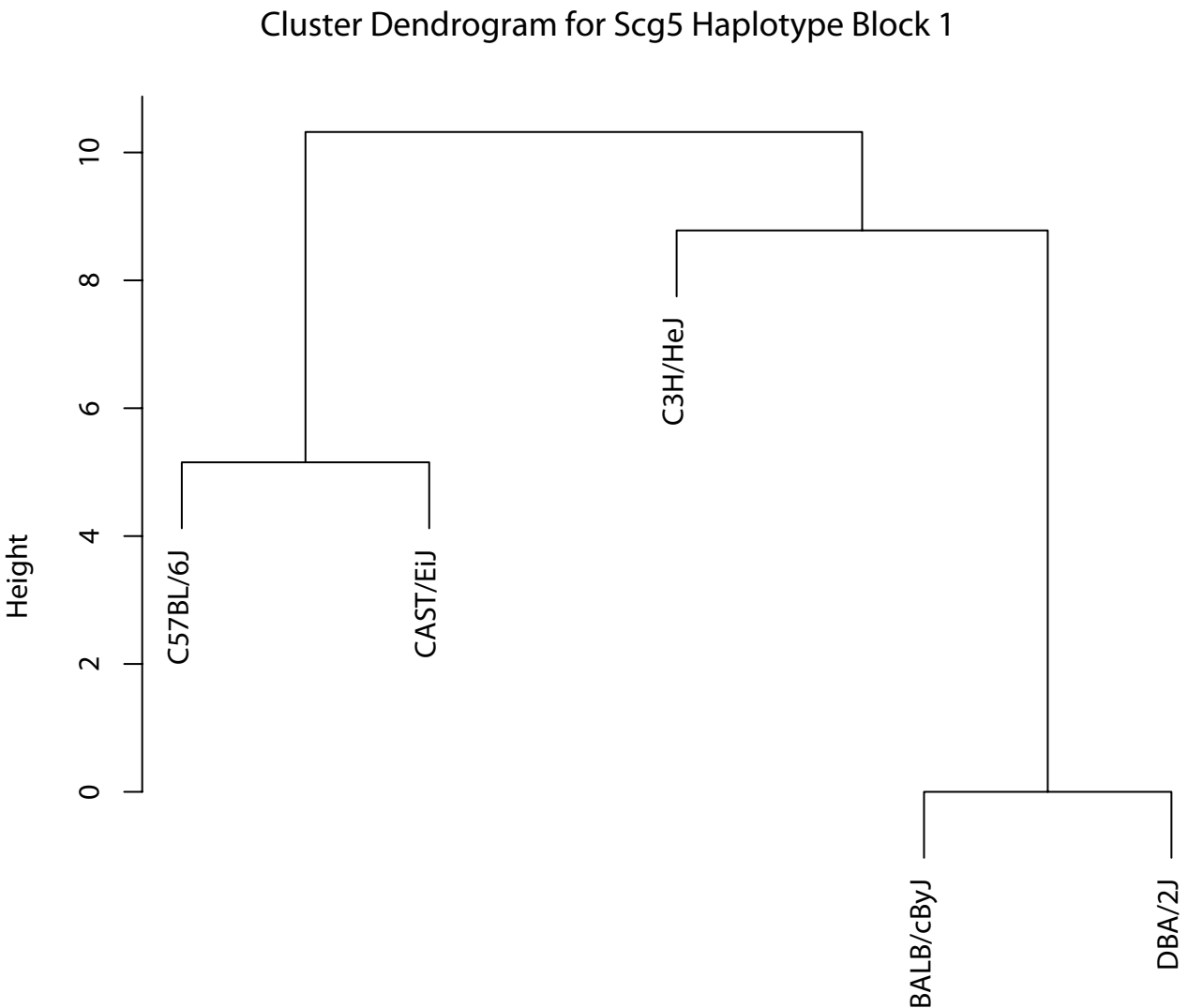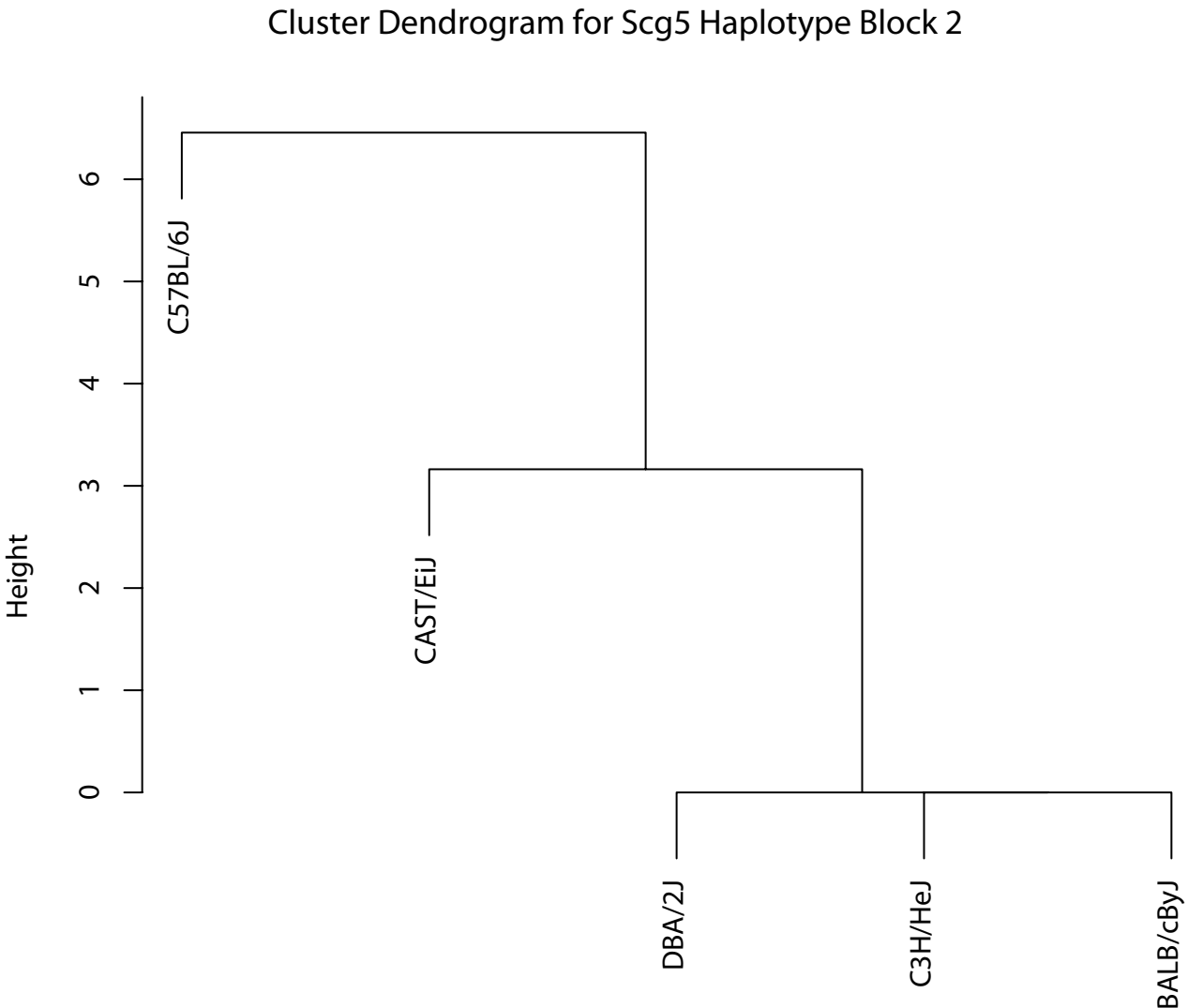

Supplement: Additional file 4 — Hierarchical clustering analysis of Scg5 haplotype blocks. Illustrates the relationships of Scg5 haplotype blocks in "low" and "high" expressing strains. [file 1471-2156-9-34-S4.pdf]
