# Supplementary material for: Overexpression of Scg5 increases enzymatic activity of PCSK2 and is inversely correlated with body weight in congenic mice
Source: BMC Genet. 2008 Apr 25;9:34. doi: 10.1186/1471-2156-9-34 (PMC2386500; doi:10.1186/1471-2156-9-34)
Supplement: Additional file 5 — Scg5 promoter sequencing primers. List of PCR primers used for Scg5 sequencing. [file 1471-2156-9-34-S5.pdf]

ADDITIONAL FILE 5. *Scg5* promoter sequencing primers

Primer	Sequence
SG1-F	AGAATTGAATTGTTAATGTACCTCTG
SG2-F	ACCTGGGCCTTCTCTGTAGG
SG3-F	CCACATTTCTTATTTGGGAGGT
SG4-F	TTTTAGTCAAGTGTACTGGCACAT
SG5-F	CCAGCAACCTATTTTTCCAA
SG6-F	CCCGAAAGTGTCCTGCAC
SG1-R	GAACATTGGAAATATAATGAACTTTTG
SG2-R	GCCCATGAAATTAGCAACAA
SG3-R	CTTGAGGAGTGCATGGGTCT
SG4-R	TCCCTGGATGCTTTCTGTTT
SG5-R	AGCCCTGGTGACAGTGCTAC
SG6-R	GATAGCTCCTGCGTCACCTC

Primer set	Amplicon Size
SG1	511
SG2	593
SG3	466
SG4	563
SG5	553
SG6	468
